# In vivo immunogenicity of Tax (11-19) epitope in HLA-A2/DTR transgenic mice: implication for dendritic cell-based anti-HTLV-1 vaccine.

**DOI:** 10.1186/1742-4690-12-S1-O28

**Published:** 2015-08-28

**Authors:** Divya Sagar, Shet Masih, Todd Schell, Steven Jacobson, Joseph D Comber, Ramila Philip, Brian Wigdahl, Pooja Jain, Zafar K Khan

**Affiliations:** 1 Department of Microbiology and Immunology, Drexel Institute for Biotechnology & Virology Research, Drexel University College of Medicine, Philadelphia, PA, USA; 2 Department of Microbiology and Immunology, The Pennsylvania State University College of Medicine, Hershey, PA, USA; 3 Viral Immunology Section, Neuroimmunology Branch, National Institutes of Health, Bethesda, MD, USA; 4 Immunotope Inc., Doylestown, PA, USA; 5 Department of Microbiology and Immunology, and the Center for Molecular Virology and Translational Neuroscience, Institute for Molecular Medicine and Infectious Disease, Drexel University College of Medicine, Philadelphia, PA, USA

## 

Viral oncoprotein Tax plays key roles in transformation of human T-cell leukemia virus (HTLV-1)-infected T cells leading to adult T-cell leukemia (ATL), and is the key antigen recognized during HTLV-associated myelopathy (HAM). In HLA-A2+ asymptomatic carriers as well as ATL and HAM patients, Tax (11-19) epitope exhibits immunodominance. Here, we evaluate CD8 T-cell immune response against this epitope in the presence and absence of dendritic cells (DCs) given the recent encouraging observations made with Phase 1 DC-based vaccine trial for ATL. To facilitate these studies, we first generated an HLA-A2/DTR hybrid mouse strain carrying the HLA-A2.1 and CD11c-DTR genes. We then studied CD8 T-cell immune response against Tax (11-19) epitope delivered in the absence or presence of Freund's adjuvant and/or DCs. Overall results demonstrate that naturally presented Tax epitope could initiate an antigen-specific CD8T cell response in vivo but failed to do so upon DC depletion. Presence of adjuvant potentiated Tax (11-19)-specific response. Elevated serum IL-6 levels coincided with depletion of DCs whereas decreased TGF-β was associated with adjuvant use. Thus, Tax (11-19) epitope is a potential candidate for the DC-based anti-HTLV-1 vaccine and the newly hybrid mouse strain could be used for investigating DC involvement in human class-I-restricted immune responses.

